# Antivaccinators

**Published:** 1901-10-19

**Authors:** 


### Antivaccinators.

The feebleness of a cause may usually be measured by the violence of the language and the terms employed by its supporters. The old argument that there was a danger of communicating disease by vaccination, which at the most was about equal to the danger of travelling in a train or crossing a street, is now entirely excluded by the use of animal lymph. Now, therefore, the anti-vaccinators are compelled to fall back upon abstract terms and statistics, the former of which appeal to the feelings, and the latter, with a little arrangement, to the reason. That specious sound, the liberty of the subject, which has taken the place of rational argument since time immemorial, and is probably responsible for more tyranny, anarchy, and bloodshed than any other term, is howled from the platform. This time it is the sacred person of the board-school child who is threatened.

No one objects to the public schools issuing notices to the parents warning them to have the boys vaccinated, or comments upon the fact that the council of almoners of Christ's Hospital have very wisely issued an order to that effect. It is only on behalf of those who are educated at the public expense that the anti-vaccinator agitates for the liberty of being a source of danger to the whole community. Apart from the general statements on abstract sociology, we are told by a barrister that "several of the most distinguished pathologists in the country were on the side of the antivaccinists, and on the other side there was absolutely no scientific knowledge on the subject," and a well-known playwright assures us that "vaccination with respect to small-pox made no difference whatever." We should be much obliged for a list of these eminent gentlemen with their professional experience and appointments stated.

We have long been accustomed to people deciding abstruse questions of politics and religion for themselves without troubling to study those subjects, nor is it at all unusual for patients to argue medicine with their doctors, but it is one thing for a person to treat his own body as he wishes, and quite another to endeavour to persuade others to disregard the advice of their technical experts, and when it comes to endangering the lives of helpless children, such action is little less than criminal.

Statistics, when simply stated, tell the truth, but little munipulation is necessary to cause them to do the reverse. No one, for instance, can deny the fact that, whereas, small-pox at the beginning of the nineteenth century, was the second most fatal disease, it is now practically the least. This is explained by the antivaccinators by the general improvement in hygiene and sanitation, but if this were the sole cause we should expect enteric, diphtheria, and many other infectious fevers would have shown the same diminution, which is not the case. Undoubtedly, bad sanitary surroundings both favour the growth of disease germs and render the human being less strong to resist their onslaught, but improved sanitation alone would have had no greater effect on the incidence of small-pox than it has on enteric and diphtheria.

Again, fatal cases of small-pox occur in those who have been vaccinated, and it may be that in some selected places as many may die as those who have not, but even if this be so we must remember that by far the greater number of people are vaccinated. If we compute these at 90 per cent. and the unvaccinated at 10 per cent., it is obvious that, even allowing an equal number of deaths, vaccination must have reduced the mortality to one-tenth what it would have been if it had not been practised. As a matter of fact the diminution is impossible to estimate, for the danger of infection does not increase in a simple but more nearly in a geometrical ratio.

